# The dynamic changes of mango (*Mangifera indica* L.) epicuticular wax during fruit development and effect of epicuticular wax on *Colletotrichum gloeosporioides* invasion

**DOI:** 10.3389/fpls.2023.1264660

**Published:** 2023-10-04

**Authors:** Jingbo Wu, Yuquan You, Xiao Wu, Feng Liu, Guoping Li, Hao Yin, Chao Gu, Kaijie Qi, Qing Wei, Songbiao Wang, Quansheng Yao, Rulin Zhan, Shaoling Zhang

**Affiliations:** ^1^ Key Laboratory of Hainan Province for Postharvest Physiology and Technology of Tropical Horticultural Products, Key Laboratory of Tropical Fruit Biology, Ministry of Agriculture, South Subtropical Crops Research Institute, Chinese Academy of Tropical Agricultural Sciences, Zhanjiang, China; ^2^ Sanya Institute of Nanjing Agricultural University, Center of Pear Engineering Technology Research, State Key Laboratory of Crop Genetics and Germplasm Enhancement, Nanjing Agricultural University, Nanjing, China; ^3^ Tropical Crops Genetic Resources Institute, Chinese Academy of Tropical Agricultural Sciences, Haikou, China; ^4^ Sanya Research Institute, Chinese Academy of Tropical Agriculture Sciences, Sanya, China

**Keywords:** mango, epicuticular wax, chemical composition, crystal morphology, different developmental periods, *Colletotrichum gloeosporioides*

## Abstract

Mango fruits are susceptible to diseases, such as anthracnose, during fruit development, leading to yield reduction. Epicuticular wax is closely related to resistance of plants to pathogenic bacterial invasion. In this study, the effect of mango fruit epicuticular wax on the invasion of *Colletotrichum gloeosporioides* was investigated, followed by to understand the changes of wax chemical composition and crystal morphology during mango fruit development using GC-MS and SEM. Results showed that the epicuticular wax of mango fruits can prevent the invasion of *C. gloeosporioides*, and ‘Renong’ showed the strongest resistance to *C. gloeosporioides*. The wax content of four mango varieties first increased and then decreased from 40 days after full bloom (DAFB) to 120 DAFB. In addition, 95 compounds were detected in the epicuticular wax of the four mango varieties at five developmental periods, in which primary alcohols, terpenoids and esters were the main wax chemical composition. Furthermore, the surface wax structure of mango fruit changed dynamically during fruit development, and irregular platelet-like crystals were the main wax structure. The present study showed the changes of wax content, chemical composition and crystal morphology during mango fruit development, and the special terpenoids (squalene, farnesyl acetate and farnesol) and dense crystal structure in the epicuticular wax of ‘Renong’ fruit may be the main reason for its stronger resistance to *C. gloeosporioides* than other varieties. Therefore, these results provide a reference for the follow-up study of mango fruit epicuticular wax synthesis mechanism and breeding.

## Introduction

Mango (*Mangifera indica* L.), an evergreen tropical fruit tree, is commonly planted in tropical and subtropical areas, including India, China, Thailand, Vietnam, Bangladesh, and Malaysia. Mango fruits are rich in vitamins A, C, and D, and sugar, dietary fiber, and some trace elements. Mango fruit is loved by people from all over the world due to its unique flavor. However, mango fruits quickly reach full maturity after harvest and are susceptible to adverse environmental factors, such as pathogen infection, thus decreasing their resistance to storage after harvest. Mangoes are processed into products, such as sauces, beverages, and dried mangoes, to reduce the effect of their intolerance during storage. However, fresh mangoes are the primary choice for consumers, so cultivating durable varieties and developing good storage methods are still good means.

Throughout their long evolutionary history, plants have developed a cuticular wax on the surface of their roots, stems, leaves, flowers, and fruits (Kim et al., 2019). The chemical composition of the cuticular wax of fruit crops has been identified at present. Hydrophobic aliphatic and cyclic chemicals, which include terpenoids, sterols, alkanes, fatty acids, primary and secondary alcohols, ketones, aldehydes, and phenols, comprise the majority of the composition of cuticular wax (Lee and Suh, 2015). Differences in wax accumulation on the cuticle of different fruit crops have been reported (Liu et al., 2021). For example, the main chemical composition in the cuticular waxes of grape (Pensec et al., 2014)and pitaya (Huang and Jiang, 2019) is triterpenoids. By contrast, the main component in the cuticular wax of guava fruit is fatty acids (Huang et al., 2020). The main component detected in the cuticular wax of citrus fruit is aldehyde (Liu et al., 2012), whereas the main compounds in the epicuticular waxes of tomato (Wu et al., 2022), pear (Wang Y. et al., 2021), and zucchini (Carvajal et al., 2021) fruit are alkanes. Similarly, differences in wax accumulation on the cuticle of different organs or developmental periods of the same crop have been reported (Guo and Jetter, 2017). Epicuticular waxes are visible or invisible coatings on the surface of plants with the naked eye, and some plants are even covered with wax powder (Pu et al., 2013). Scanning electron microscopy (SEM) has been used to characterize the epicuticular wax microstructure of fruits. Wax crystal morphology can be broadly categorized as rod, platelet, film, granular, lamellar, and so on (Engel and Barthlott, 1988; Barthlott et al., 1998), and the crystal morphology of epicuticular wax is closely related to chemical composition (Chu et al., 2017).

Previous studies have shown that cuticular wax can help plants resist UV radiation and fungal invasion, reduce non-stomatal water loss, and maintain mechanical strength (Yeats and Rose, 2013). For example, cuticular wax has been reported to help reduce water transpiration in pepper (Parsons et al., 2012), lily (Cheng et al., 2021), and mulberry (Sajeevan et al., 2017); moreover, in blueberry (Miles and Schilder, 2013), pear (Zhang et al., 2023) and grape (Özer et al., 2017) fruits, cuticular wax is related to disease resistance. Mango is susceptible to anthracnose during development and after harvest, thus reducing the economic benefits. However, the epicuticular wax of mangoes is limited to the observation of crystal morphology, and research on its chemical composition and interaction with adverse environments is limited (Prinsloo et al., 2004). According to early field observations in the laboratory, resistance to anthracnose differed among four varieties, ‘Renong,’ ‘Guiqi,’ ‘Irwin’ and ‘Dashehari’, and different developmental periods. In the present work, the changes in epicuticular wax crystal morphology and chemical composition of mango fruit throughout its developmental period was investigated. In addition, the effect of mango cuticular wax on the disease was investigated by inoculating with *C. gloeosporioides*. This work could provide a certain reference basis for future research on the synthesis and function of mango epicuticular wax.

## Materials and methods

### Plant materials

The fruits of ‘Renong’ (‘Renong No. 1’), ‘Guiqi’ (‘Guire No. 82’), ‘Irwin’ and ‘Dashehari’ used in the experiment were harvested from the mango picking garden of Guangqian Famous Fruit Company in Suixi County, Zhanjiang City, Guangdong Province. The mango fruits at five developmental periods, namely, 40 days after full bloom (DAFB), 60 DAFB, 80 DAFB,100 DAFB, and 120 DAFB, were utilized in this study. Fifteen mango fruits were harvested at any developmental period to identify the chemical composition and crystal morphology of epicuticular wax. In addition, at 120 DAFB, an additional sixty fruits were harvested from ‘Renong,’ ‘Guiqi,’ ‘Irwin’ and ‘Dashehari’ for *C. gloeosporioides* inoculation. The harvested fruits were relatively uniform in size and maturity, with no damage to the fruit surface. The harvested fruits were wrapped in foam bags, placed in boxes, and transported to the South Subtropical Crop Research Institute of Chinese Academy of Tropical Agricultural Sciences.

### SEM observations

The crystal morphology observation of mango epidermal waxes was referred to the method of Trivedi et al. (2019). In each period, mango fruits with intact appearance were selected, and a square piece of peel (4 mm × 4 mm) was cut near the equator of the fruits by using a surgical blade. The peels were placed in a 2.5% glutaraldehyde solution for 2 h and then dried using a critical point desiccator (Quorum, USA) when the dehydration was complete. The dried pericarp was treated with gold spraying using an ion sputterer (Hitachi, Japan), and then the epicuticular wax microstructure of these treated pericarps was observed on a Hitachi Regulus 8100 (Japan).

### Extraction of cuticular waxes and determination of wax content

The wax was extracted using chloroform immersion. Nine fruits were divided into three groups, sequentially placed in a beaker, and soaked in chloroform for 2 min. Then, 2 µl n-tetracosane solution (10 mg/mL) was added and dried at room temperature by using a slow nitrogen airflow. The fruit wax content was determined by calculating the fruit’s surface area (Sa) in relation to the Sa of an irregular spheroid (Chen et al., 2002) as follows: wax content = (W1 - W0)/Sa, where W1 is the weight of the vials and wax (μg), W0 refers to the initial weight of the vials (μg), and Sa refers to the surface area of the nine fruits.

### GC-MS analysis

The wax extract was derivatized by mixing 200 µL bis-*N,O*- (trimethylsilyl)trifluoroacetamide (Solarbio) and 200 μL pyridine (Sigma-Aldrich) into the wax extract and reacting at 70°C for 1 h. After the reaction was complete, the solution was blown dry with slow stream of nitrogen at room temperature. Then, the dried wax-derived compound was redissolved in 1 mL chromatography-grade chloroform. Afterwards, the mixture was filtered through a 0.45 µm filter by using a 1 mL syringe into a 2 mL brown feed vial with the use of a cap and a sealant. The vial was kept in a refrigerator at -80°C after being taped shut at the point where the top meets the vial.

Gas chromatography–mass spectroscopy (GC-MS, Bruker 450-GC and Bruker 320-MS) was used to examine the wax fractions. Samples were fed into the instrument through a capillary column (BR-5MS, length 30 m, inner diameter 0.25 μm, 0.25 μm film thickness) by using 1.2 mL/min of helium as the carrier gas. The following parameters were employed during the GC–MS analytical process: the temperature of the transmission line was 280°C, the inflow port temperature was 280°C, the ion-source temperature was 250°C, the quadrupole temperature on GC-MS was 150°C, the electron energy (EI) was 70 eV, and the MS scan range was 50–650 m/z. The instrument was operated at 50°C for 2 min following the GC-MS inhalation of the sample and then at 200°C at a rate of 40°C/min for 2 min. After 30 min, 320°C was reached by ramping up from 200°C at a rate of 3°C/min.

### Colletotrichum gloeosporioides inoculation

Sixty mature mango fruits were evenly divided into two groups. One group was treated with blue butyl rubber to destroy the wax on the fruit surface as the treatment group, and the other group was not treated as the control group. Each fruit was inoculated with two points, one of which was inoculated with *C*. *gloeosporioides* spore suspension (1 × 10^6^ spores/mL of solution) onto the fruit surface, and the other spot was inoculated with sterile culture solution as a control. The fruits were then placed flat in a freshness box, cotton soaked in sterile distilled water, and placed in a preservation box to retain humidity. The test fruits were kept at room temperature and checked daily for disease incidence. The diameter of the spots (mm) was measured when the incidence of the participating types reached 100% after inoculation.

### Statistical analysis

Principal component analysis (PCA) was plotted using SIMCA version 14.1. GraphPad Prism 9 software was used to create bar graphs, Heatmaps were created with TBtools and correlation analysis was conducted in Origin 2023. SPSS version 23.0 software was used for significant difference analysis, and different letters on each column indicated significant difference at 5% level. For each batch of data, three replicates were carried out, and the data were expressed as mean ± SD.

## Results

### Effect of mango epicuticular waxes on *C. gloeosporioides* infection

**Figure 1:**
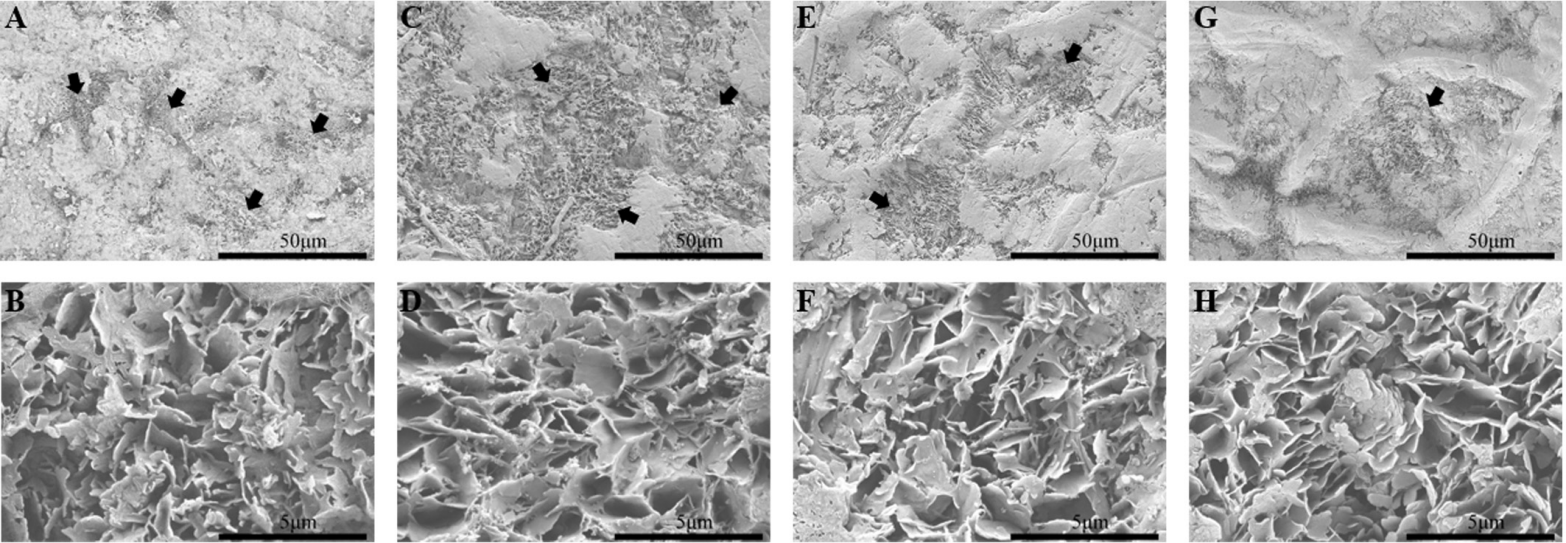
Wax microstructure of four mango fruits after dewaxing at 120 DAFB. **(A, B)** ‘Irwin’; **(C, D)** ‘Renong’; **(E, F)** ‘Guiqi’; **(G, H)** ‘Dashehari’. Among, 50 μm represents a magnification of 1000 times and 5 μm represents a magnification of 10,000 times. The black arrow points to where the wax layer has been damaged.

**Figure 2:**
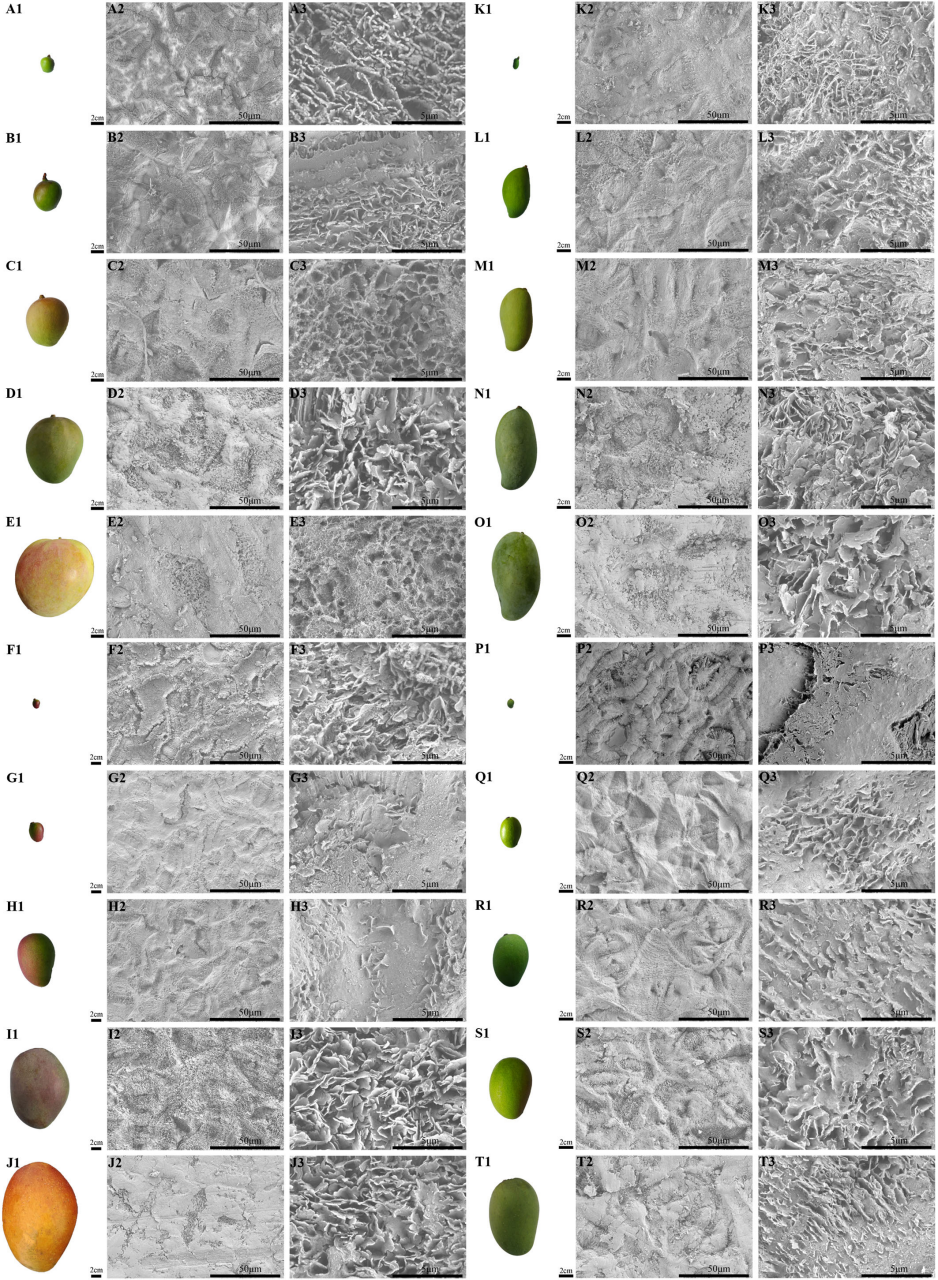
The fruit morphology and magnification series of SEM images of the cuticular wax of developing ‘Renong’, ‘Irwin’, ‘Guiqi’ and ‘Dashehari’ mango fruit. (A1-E1, F1-J1, K1-O1, P1-T1) are fruit shapes of developing ‘Renong’, ‘Irwin’, ‘Guiqi’ and ‘Dashehari’ mango fruit, respectively. On the right side of the fruit is the corresponding epicuticular wax microstructure. Scale bars = 50 μm, magnification = 1000×; scale bars = 5 μm, magnification = 10,000×.

The SEM results showed that the mango fruits treated with blue butyl gum cannot completely remove the epicuticular wax, did not show any change in crystal morphology, and destroyed the integrity of the epicuticular wax (**Figures 1**, **2**). The inoculation test of *C*. *gloeosporioides* showed that the incidence of ‘Irwin’ and ‘Dashehari’ fruits in the control group and the dewaxed treatment group reached 100% after 2 d of inoculation, while the incidence of ‘Renong’ and ‘Guiqi’ after dewaxed treatment was 36.67% and 60.00%, which was 1.38 and 1.29 times that of the control group (**Table 1**). After 6 d of inoculation, the disease index of ‘Irwin’ fruit after dewaxing treatment could not be counted due to decay, while the disease index of ‘Renong,’ ‘Guiqi’ and ‘Dashehari’ after dewaxing treatment were 24.51, 58.57 and 89.48, respectively, which were 1.19, 1.14 and 1.57 times that of the control group. However, there was no significant difference in the disease index between the control group and the dewaxing treatment group of ‘Renong’ and ‘Guiqi’.

**Table 1:** Incidence and disease index of mango intact and dewaxed fruits after inoculation with *C. gloeosporioides*.

Variety	Control (intact)		Treatment (dewaxed)	
	Two-day incidence of inoculation	Disease index	Two-day incidence of inoculation	Disease index
Renong	26.67%	20.67 d	36.67%	24.51 d
Guiqi	46.67%	51.39 c	60.00%	58.57 c
Deshehari	100.00%	57.17 c	100.00%	89.48 a
Irwin	100.00%	73.92 b	100.00%	–

Different letters indicate significant differences at P < 0.05.

### Epicuticular wax load

**Figure 3:**
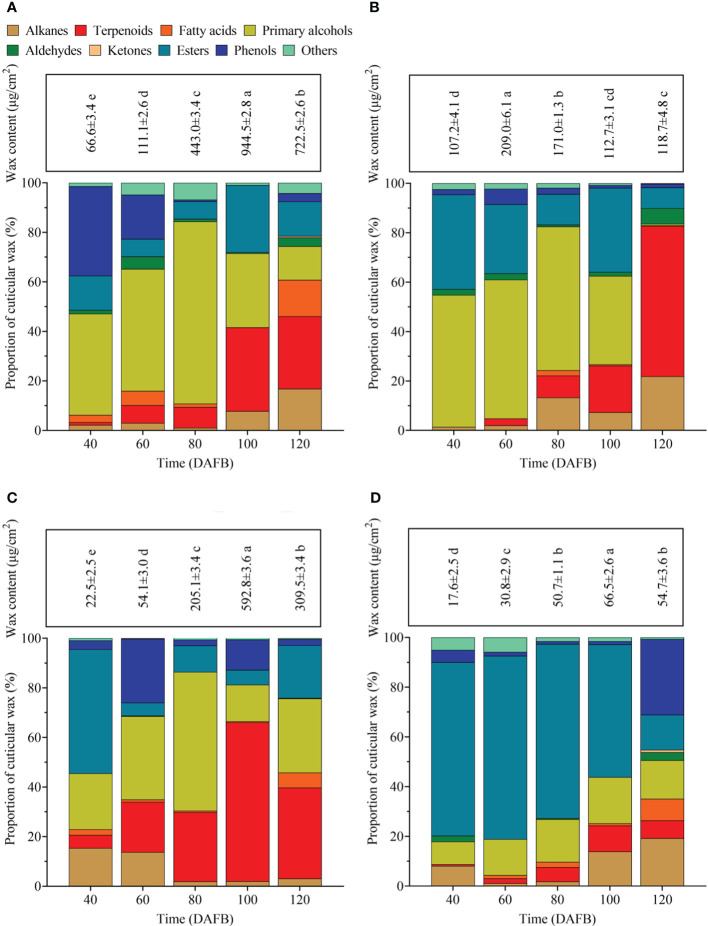
Total epicuticular wax concentrations and relative wax compositions in **(A)** ‘Renong,’ **(B)** ‘Irwin,’ **(C)** ‘Guiqi,’ and **(D)** ‘Dashehari’ fruits at five developmental stages (20, 40, 60, 80 and 120 DAFB). Data are mean ± SD (n=3) and letters shown above each bar indicate significant differences at P<0.05.

During the entire developmental period, the epicuticular wax content of ‘Renong,’ ‘Guiqi’ and ‘Dashehari’ showed a trend of first increasing and then decreasing, with the minimum and maximum values appearing at 40 and 100 DAFB, respectively (**Figures 3A, C, D**). Meanwhile, the epicuticular wax content of ‘Irwin’ first increased and then decreased from 40 DAFB to 100 DAFB, and then it slightly decreased at 120 DAFB, which was no significant difference compared with the previous period, with the minimum and maximum values appearing at 40 and 60 DAFB, respectively (**Figure 3B**). Throughout the developmental period, the cuticular wax content of ‘Dashehari’ was the lowest among all varieties. However, from 40 DAFB to 60 DAFB, the cuticular wax content of ‘Irwin’ was higher than that of other varieties, and from 80 DAFB, the cuticular wax content of ‘Renong’ was the highest. At 120 DAFB, the cuticular wax contents of ‘Renong’ were 5.09, 1.33, and 12.20 times higher than those of ‘Irwin,’ ‘Guiqi’ and ‘Dashehari’, respectively.

### Composition of epicuticular wax

A total of 95 compounds were detected in the epicuticular waxes of the four mango varieties during five developmental periods (**Supplementary Table S1**). These compounds can be classified into nine categories: primary alcohols, alkanes, aldehydes, fatty acids, ketones, esters, phenols, terpenoids, and some other compounds (**Figure 3**). The chemical composition in the epicuticular wax varied in terms of developmental time and mango fruit variety. For example, aldehydes were detectable throughout the developmental period in the epicuticular wax of ‘Renong’ and ‘Irwin’, while ‘Guiqi’ only showed aldehydes at 60 DAFB. Meanwhile, ‘Dashehari’ did not show aldehydes at 60 and 100 DAFB. Moreover, primary alcohols were the main compound in the epicuticular wax of ‘Renong,’ ‘Irwin’ and ‘Guiqi’ at the early developmental periods, whereas terpenoids were the main compound at the late developmental periods. Esters were the main compounds in the epicuticular wax of ‘Dashehari’ throughout the developmental period.

**Figure 4:**
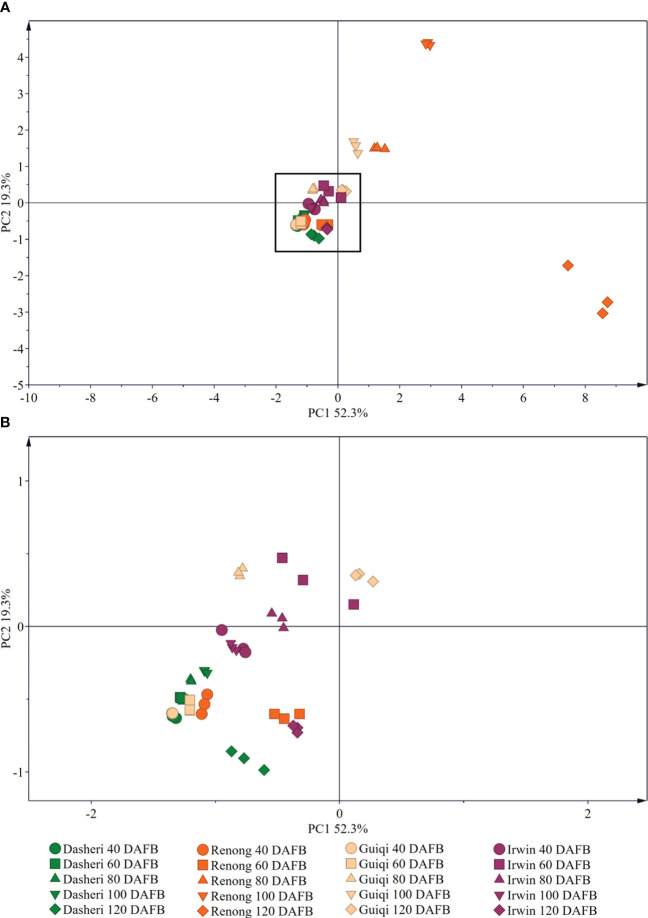
Principal component analysis (PCA) of the chemical compositions of epicuticular wax in four mango fruit cultivars during all development stage. **(B)** is a black box locally enlarged graph in **(A)**.

The PCA results showed that the three principal components PC1 and PC2 were 52.3% and 19.3%, respectively, which can explain 71.6% of the variation (**Figure 4**), and there were differences in the epicuticular wax of ‘Renong,’ ‘Guiqi’ and ‘Dashehari’ mango varieties with fruit development. There were significant differences in the cuticular wax content and components of ‘Irwin’ fruit at 60, 80 and 120 DAFB compared with those at 40 and 100 DAFB, but there was no significant difference in the cuticular wax content and components at 40 and 100 DAFB. In addition, there were also differences in fruit epidermal wax between different mango varieties at the same period. Among them, the samples of ‘Guiqi’ and ‘Dashehari’ were coincident at 40 and 60 days after flowering, indicating that the fruit wax was significantly different, but it was significantly different from ‘Renong’ and ‘Irwin’. However, from 80 DAFB, different mango varieties could be clearly distinguished in the same period, indicating that there were significant differences in fruit epicuticular wax.

### Aliphatic compounds

Primary alcohols, alkanes, fatty acids, aldehydes, and ketones were the aliphatic compounds detected in the epicuticular wax of mango fruit (**Figure 3**). With fruit development, the change trend of aliphatic compound content in the epicuticular wax of different mango varieties differed, among which ‘Renong’ and ‘Irwin’ showed a trend of first increasing and then decreasing. Meanwhile, the change trend of ‘Guiqi’ from 40 DAFB to 100 DAFB was similar to that of the above varieties, and then it increased at 120 DAFB (**Supplementary Table S1**). The content of aliphatic compound in the cuticular wax of ‘Dashehari’ gradually increased with fruit development compared with that in other varieties. Moreover, this content in ‘Dashehari’ was the lowest at any developmental period.

**Figure 5:**
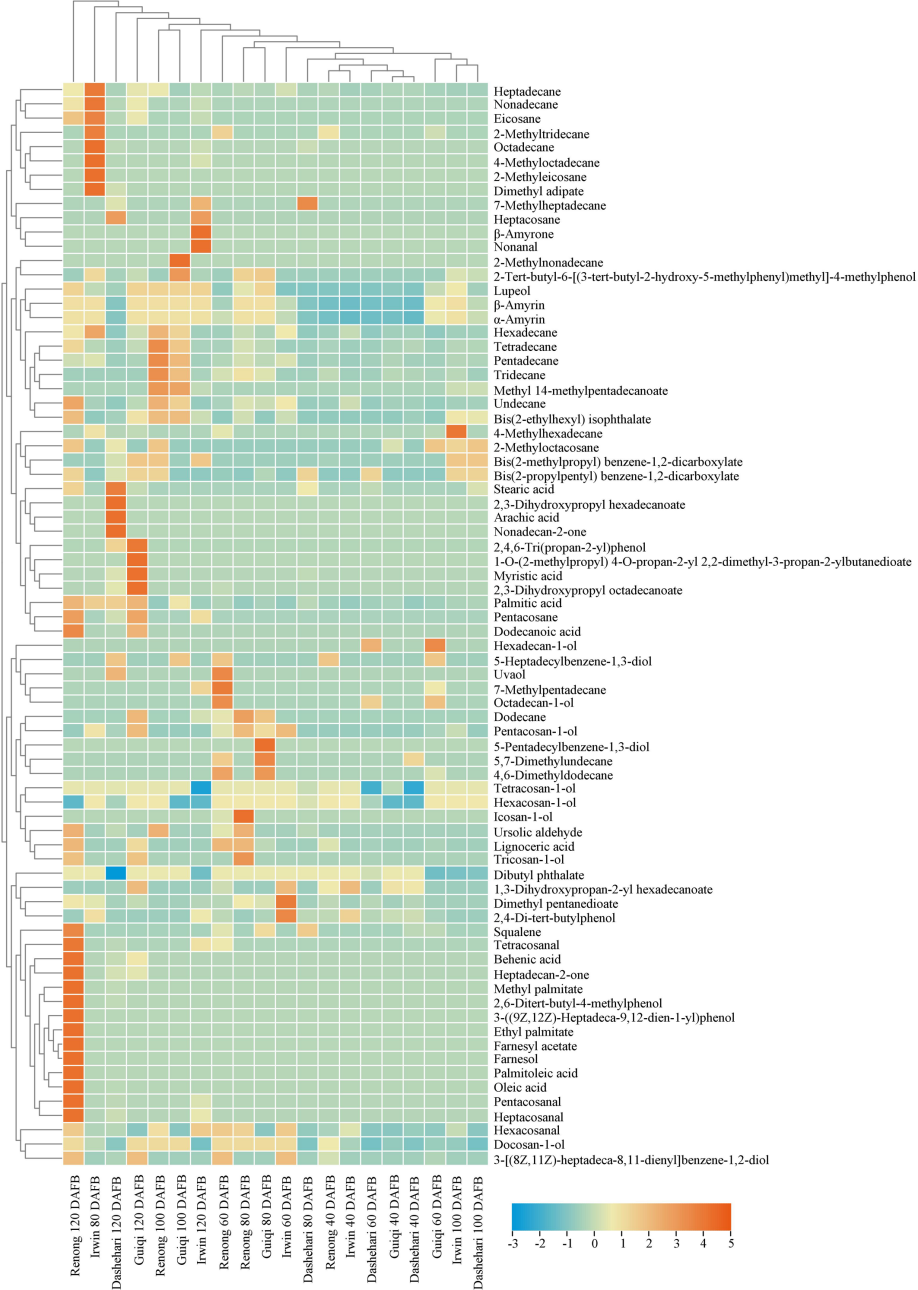
Heatmap of alkanes, fatty acids, primary alcohols, ketones, aldehydes, terpenoids, esters and phenols in the epicuticular wax of ‘Renong’, ‘Irwin’, ‘Guiqi’ and ‘Dashehari’ fruits at 20, 40, 60, 80 and 120 DAFB. Colors indicate chemical composition levels.

The content of primary alcohols in the epicuticular wax of different mango varieties showed different trends throughout the developmental period, with ‘Renong,’ ‘Irwin’ and ‘Dashehari’ showing a trend of first increasing and then decreasing (**Supplementary Table S1**). ‘Guiqi’ showed a similar trend from 40 DAFB to 100 DAFB as the abovementioned varieties, but then its content increased slightly at 120 DAFB. The primary alcohol content in the epicuticular wax of ‘Dashehari’ remained the lowest at any developmental period. In addition, the primary alcohol content in the cuticle wax of ‘Irwin’ was higher than that in other three species at 40 and 60 DAFB. The highest primary alcohol content was found in the epidermal wax of ‘Renong’ from 80 DAFB onwards. Tetracosan-1-ol was determined to be the main primary alcohol in the epicuticular wax of the four mango varieties throughout the entire developmental period (**Figure 5**).

High contents of fatty acids were detected in the epicuticular wax of the four mango varieties at 120 DAFB (**Supplementary Table S1**). The fatty acid content of ‘Renong’ was 0.06, 4.55, and 17.93 times higher than those of ‘Irwin,’ ‘Guiqi’ and ‘Dashehari’, respectively. At this time, palmitic acid was the main fatty acid in all the varieties. However, oleic and palmitoleic acids were detected only in the cuticular wax of ‘Renong’ and ‘Dashehari’ (**Figure 5**).

Alkanes were the most abundant compound in the epicuticular wax of mango fruit. A total of 22 alkane compounds were detected at five developmental periods (**Supplementary** **Table S1**). At 100 and 120 DAFB, the alkane content in the epicuticular wax of ‘Renong’ was significantly higher than that in ‘Irwin,’ ‘Guiqi’ and ‘Dashehari’. In addition, hexadecane was detected in the epicuticular wax of all four mango varieties at five developmental periods (**Figure 5**). Pentacosane was only detected in the cuticular wax of all four varieties at 120 DAFB, and heptacosane was only found in ‘Irwin’ and ‘Dashehari’.

Aldehydes were detected at low contents in the epicuticular wax of ‘Guiqi’ and ‘Dashehari’ during the entire developmental period. In addition, the aldehyde content in the epicuticular wax of “Irwin” was higher than that of other varieties at 40 DAFB, and from 60 DAFB, the aldehyde content in ‘Renong’ was higher than that in other varieties (**Supplementary Table S1**). Hexacosanal was the main aldehyde in the cuticular wax of ‘Renong,’ ‘Irwin’ and ‘Dashehari’ at 120 DAFB, whereas ‘Guiqi’ only showed hexacosanal at 60 DAFB (**Figure 5**).

Ketones were detected only in the epicuticular wax of ‘Renong,’ ‘Guiqi’ and ‘Dashehari’ at 120 DAFB, with the contents of 4.17, 0.68, and 0.52 µg/cm^2^, respectively (**Supplementary Table S1**). However, no ketones were found in the epicuticular wax of ‘Irwin’ at any developmental period. In addition, heptadecan-2-one was the major ketone in the epicuticular wax of ‘Renong’ and ‘Guiqi,’ and nonadecan-2-one and heptadecan-2-one were detected in the epicuticular wax of ‘Dashehari’ (**Figure 5**).

### Esters and phenols

Esters, specifically aromatic esters and fatty esters, were detected in the fruit epicuticular wax of the four mango cultivars during development. The ester content in the epicuticular wax of ‘Renong’ was higher than that of other varieties at 100 DAFB, while ‘Irwin’ had the highest content in other periods (**Supplementary Table S1**). GC-MS showed that dibutyl phthalate was the most abundant ester in the epicuticular wax of the four mango varieties. In addition, bis(2-propylpentyl)benzene-1,2-dicarboxylate was detected in the epicuticular wax of ‘Renong’ at 100 DAFB, with the highest content in five developmental periods (**Figure 5**).

Phenol was an aromatic compound detected in the epicuticular wax of mango. Compared with other varieties, ‘Renong’ did not show phenols at 100 DAFB (**Supplementary Table S1**). Meanwhile, 2-4-di-tert-butylphenol was the most abundant phenolic compound in the fruit epicuticular wax of all varieties during the five developmental periods. In addition, 3-((9*Z*,12*Z*)-heptadeca-9,12-dien-1-yl)phenol was detected only in the epicuticular wax of ‘Renong’ at 120 DAFB, and 5-pentadecylbenzene-1,3-diol was detected only in the epicuticular wax of ‘Guiqi’ at 80 DAFB (**Figure 5**).

### Terpenoids

Terpenoids were the main cyclic compounds in the epicuticular wax of mango throughout the entire developmental period (**Figure 3**). The content of terpenoids in the wax of ‘Renong,’ ‘Guiqi’ and ‘Dashehari’ showed a trend of first increasing and then decreasing (**Supplementary Table S1**). However, the content of terpenoids in the epicuticular wax of ‘Irwin’ gradually increased from 60 DAFB. Compared with other varieties, ‘Dashehari’ had a low content of terpenoid at any developmental period. From 40 DAFB to100 DAFB, the content of terpenoids in the epicuticular wax of ‘Guiqi’ was higher than that of other varieties. The content was higher in ‘Renong’ than in other varieties at 120 DAFB.

The GC-MS results showed that seven terpenoids were detected in the epicuticular wax of mango fruit (**Figure 5**; **Supplementary Table S1**). β-Amyrin and α-amyrin were the main terpenoids in the wax of mango fruit at any developmental period. Lupeol was the most abundant terpenoid in the wax of ‘Renong’ and ‘Guiqi’ fruit throughout the entire developmental period. In addition, some terpenoids that only exist in specific varieties were observed. In particular, uvaol was detected in the wax of ‘Renong’ and ‘Dashehari’ and β-amyrone was detected in the wax of “Irwin.” By contrast, squalene was not detected in the epicuticular wax of ‘Irwin,’ and ursolic aldehyde was not detected in the wax of ‘Irwin’ and ‘Guiqi’ at any developmental period.

### Change in waxy crystal morphology during all developmental periods

From 40 DAFB to 120 DAFB, the surface of the four varieties gradually became rough, where a white coating was attached to the surface (**Figure 3** A1-E1; F1-J1; K1-O1; P1-T1). The SEM results showed that epicuticular wax can be observed at any developmental period, and differences were found in the microscopic morphology of the waxes among different developmental periods and cultivars. From 40 DAFB to 80 DAFB, new waxy crystals were gradually formed on the surface of ‘Renong,’ ‘Irwin’ and ‘Guiqi’ and ‘Dashehari’ fruits, and the size and density of the crystals gradually increased (**Figure 3** A2-C2; A3-C3; F2-H2; F3-H3; K2-M2; K3-M3; P2-R2; P3-R3). The adjacent waxy crystals were bonded to each other to form a larger waxy structure. With the further development of the fruit (from 100 DAFB to 120 DAFB), the density and size of the crystals covered on the surface further increased, and the adjacent crystals adhered to each other (**Figure 3** D2-E2; D3-E3; I2-J2; I3-J3; N2-O2; M3-O3; S2-T2; S3-T3). Furthermore, the surface of mango fruit is mainly covered with irregular platelet wax crystals arranged in different directions.

## Discussion

### Changes in epicuticular wax accumulation during mango fruit development

In this experiment, the epicuticular wax content of mango generally increased and then decreased with fruit development (**Figure 3**). This result was also observed on blueberries (Cao et al., 2022) and grapes (Arand et al., 2021). However, bilberry (Trivedi et al., 2021) and ‘Newhall’ orange (Wang et al., 2016) differ from mangoes in terms of the changes in cuticular wax content with fruit development. A notable detail that the amount of wax per mango gradually increased with fruit development, whereas the epicuticular wax content of mango fruit showed a trend of first increasing and then decreasing. Therefore, this difference may be due to the fact that the deposition rate of epicuticular wax is much lower than the rate at which the surface area of mango fruits increases; the exact reason remains to be verified (Wu et al., 2019).

The GC-MS results indicated that the proportion of various chemical compositions in the epicuticular wax of mango fruit changed with fruit development, and PCA results also confirmed this result (**Figures 3**, **4**). From 40 DAFB to 80 DAFB, the chemical composition of the epicuticular wax of ‘Renong,’ ‘Irwin’ and ‘Guiqi’ was dominated by primary alcohols. When the fruits developed at 100 or 120 DAFB, terpenoids became the main compounds in the epicuticular wax. Similar phenomena were observed in the epicuticular wax of blueberry (Cao et al., 2022), pear (Wu et al., 2019), and citrus (Wang et al., 2016) fruits throughout their developmental period. On the contrary, the dominant compounds in the cuticular wax of grape (Zhang et al., 2021) and bilberry (Trivedi et al., 2021) remained unchanged as the fruits developed. Moreover, in contrast to other varieties, the epicuticular wax of ‘Dashehari’ was dominated by esters throughout the developmental period, which is different from the phenomenon observed for grapes (Zhang et al., 2021). In conclusion, the differences in the chemical composition of epicuticular waxes between different developmental periods and mango varieties may be due to varietal characteristics and environmental factors. The exact causes need to be verified. In the present study, the content of primary alcohol detected in the cuticular wax of ‘Renong,’ ‘Irwin’ and ‘Dashehari’ showed a trend of increasing first and then decreasing with fruit development. Similar results were observed in pear (Wu et al., 2019) and wheat (Wang et al., 2015). In addition, the present study revealed that ‘Renong,’ ‘Guiqi’ and ‘Dashehari’ contained higher levels of terpenoids in the cuticular wax throughout fruit development, and the same results were found in bilberry (Trivedi et al., 2021) and blueberry (Cao et al., 2022). By contrast, ‘Irwin’ showed a gradual increase in terpenoid content with fruit development, and similar results were found in grapes (Zhang et al., 2021). Furthermore, the differences in chemical composition of the epicuticular wax between different developmental periods and mango varieties could provide some reference for the subsequent investigation of the functions of different chemical compositions in epicuticular wax and the mechanism of epicuticular wax synthesis in mango.

The carbon chain length of the primary alcohol compounds detected in the epicuticular wax of mango was C16-C26. Similar results were found in ‘Korla’ pear (Wang P. et al., 2021) and goji (Wang et al., 2021). Likewise, alkanes were aliphatic compounds found in the epicuticular wax of mango fruits at different developmental periods, and the alkane carbon-chain lengths were dominated by short chains (C11-C17), as shown in **Supplementary Table S1**. However, the distribution of alkane carbon chains in the cuticular wax of pear (Wu et al., 2018), citrus (Romero and Lafuente, 2022), and cherry (Gutiérrez et al., 2021) fruits was detected from C21 to C34. Furthermore, C24-C27 very-long-chain fatty aldehydes can be detected in the epicuticular waxes of mango fruits at different developmental periods, whereas only C16 and C18 long-chain fatty aldehydes were found in the cuticular wax of pear fruits (Wu et al., 2019). Terpenoids were among the compounds with high contents in the epicuticular wax of mango fruit, with α-amyrin, β-amyrin, and lupeol being the main terpenoids. In addition, ursolic aldehyde and uvaol were only detected in the epicuticular wax of ‘Renong’ and ‘Guiqi’, and β-amyrone was only found in the cuticular wax of ‘Irwin’ at 120 DAFB. In conclusion, the chemical composition of mango fruit epicuticular wax varies among different varieties and developmental stages, and the mechanism of these differences needs to be explored.

### Crystal morphology of epicuticular wax in mango

Previous studies have shown that the epicuticular wax of mango is structurally divided into two layers, the outer layer with a certain morphology of wax crystals and the inner amorphous wax film (Prinsloo et al., 2004). Bally (1999) found that the surface wax microstructure of ‘Kensington Pride’ mango fruit showed dynamic changes during fruit development. In this study, the size and density of waxy crystals covered on the surface of ‘Renong,’ ‘Irwin,’ ‘Guiqi’ and ‘Dashehari’ gradually increased with the development of fruit, and the adjacent waxy crystals adhered to each other. At 100 and 120 DAFB, the surface of mango fruit was covered with irregular platelet-like waxy crystals arranged in different directions. Similar phenomena were observed in the epicuticular waxes of ‘Kyoho’ and ‘Red Globe’ throughout the developmental period (Zhang et al., 2021). In addition, a wax ridge structure was also observed on the ‘Renong,’ ‘Irwin,’ ‘Guiqi’ and ‘Dashehari’ fruit surface, consistent with the results observed earlier on the ‘Keitt’ mango fruit (Tafolla-Arellano et al., 2017).

The morphology of cuticular wax crystals is closely related to their chemical composition. In this study, primary alcohols and terpenoids were the dominant compounds in the fruit epicuticular wax of ‘Renong,’ ‘Irwin’ and ‘Guiqi’ during the entire developmental period. Therefore, the high content of primary alcohols and terpenoids in the wax is the main reason for the formation of irregular platelet wax crystals, consistent with the results in Poaceae plants (Koch et al., 2006), *Sedum rupestre* (Stevens et al., 1994), and bilberry (Trivedi et al., 2021). However, the high content of terpenoids in the epicuticular wax of grape fruit formed lamellar crystals (Zhang et al., 2021), and the irregular platelet crystal morphology attached to the waxy surface of pear (Wu et al., 2017) and citrus (Wang et al., 2014) fruits was found to be related to the high content of alkanes. In addition, ketones were detected in the chemical composition of the epicuticular wax of ‘Renong,’ ‘Guiqi’ and ‘Dashehari’ fruits at 120 DAFB, but no rod-like crystals were observed, which was different from previous studies (Barthlott et al., 1998; Trivedi et al., 2021). Therefore, the structure of plant cuticular wax is not only affected by chemical components, but may also be affected by other factors. Previous studies have shown that the wax structure of plant surface is also affected by the crystallinity and polarity of cutin, which affects the orientation and preferred direction of crystal growth on plant surface (Koch et al., 2006; Wang P. et al., 2021). Additionally, an uncommon filamentous structure was found on the microscopic morphology of the epicuticular wax of ‘Renong’ at 120 DAFB (**Figure 2** J2, J3), which is speculated to be a special crystal structure of the epicuticular wax of mature ‘Renong’. This structure could increase the crystal density of the epicuticular wax to a certain extent, but its role remains to be explored.

### Prevention of *C. gloeosporioides* invasion by the epicuticular wax of mango fruit

Anthracnose disease in mango, generally caused by *C. gloeosporioides*, brings great damage to the production of mango, especially for susceptible mango cultivar (Arauz, 2000). Previous studies have pointed out that plant cuticular wax plays a crucial role in the resistance to pathogenic bacteria (Gniwotta et al., 2005). Sorghum leaf epicuticular wax can inhibit the growth of *Colletotrichum sublineola* (Xiong et al., 2023). The removal of the epicuticular wax layer of *Hordeum chilense* leaves makes it easier for the appressoria of *Puccinia hordei* to grow on the stomata (Vaz Patto and Niks, 2001). Ni et al. (2014) found that the resistance of resistant *Brassica napus* leaves to *Sclerotinia sclerotiorum* decreased after the removal of epicuticular wax, while the susceptible varieties did not change significantly. In this experiment, when the epicuticular waxes were injured from mango fruit, ‘Dashehari’ developed more bigger spots, resulting in a higher disease index compared to fruits with wax intact, while no significant change was observed for ‘Renong’ and ‘Guiqi’ (**Table 1**). This might have been caused by the high content of fatty acids, primary alcohols, and esters in the surface wax of ‘Renong’ and ‘Guiqi’. It is reported that plant cuticular wax contains many antifungal compounds (Özer et al., 2017). Terpenoids are naturally occurring active substances in cuticular wax, and they have an effect on fungal invasion (Oliveira Lino et al., 2020). Moreover, terpenoids in the cuticular wax of goji berry (Wang P. et al., 2021) and pear (Yin et al., 2011) fruits play an important role in resisting the infection of *Alternaria* rot. Squalene in cuticular wax has antifungal activity and can inhibit the growth of pathogens (Wu et al., 2017). Our results showed that ‘Renong’ had strongest resistance to *C. gloeosporioides* compared to other varieties. The SEM showed that the wax layer covered on the surface of ‘Renong’ fruit at 120 DAFB contained some special filamentous structures, which made the wax structure denser. Moreover, squalene, farnesol and farnesyl acetate were only detected in the epicuticular wax of ‘Renong’ fruit at 120 DAFB. Taken together, we speculate that the observation of strongest resistance to *C. gloeosporioides* in ‘Renong’ may be attributed to it having the special terpenoids in cuticular wax, particularly squalene, farnesyl acetate and farnesol, dense wax crystal structure. Whether the chemical components of mango fruit cuticular wax play a role in the invasion of *C. gloeosporioides*, it is necessary to separate each component for verification.

## Conclusion

In this study, the epicuticular wax content of four mango varieties generally increased and then decreased with fruit development. The GC-MS results showed that a total of 95 compounds were detected in the epicuticular wax of the four mango varieties at five developmental periods. The chemical components of the epicuticular wax of ‘Renong,’ ‘Irwin’ and ‘Guiqi’ were mainly primary alcohols from 40 DAFB to 80 DAFB, and then they turned into terpenes at 100 and 120 DAFB. However, the chemical composition in the epicuticular wax of ‘Dashehari’ during the whole developmental period were mainly esters. Moreover, During the development of mango fruit, the density and size of wax crystals covered on the surface gradually increased, and the adjacent wax crystals adhered to each other, and the irregular platelet-like wax crystals were the main wax structure. Furthermore, Mango fruit epicuticular wax can play a key role in resisting *C. gloeosporioides*, and the reason why ‘Renong’ has stronger resistance to anthracnose than other varieties may be that the fruit epicuticular wax is rich in squalene, farnesol and farnesyl acetate, and dense wax crystal structure. This study could provide some reference for studying the mechanism of epicuticular wax synthesis in mango fruits and developing methods for the control of anthracnose before and after harvest.

## Data availability statement

The original contributions presented in the study are included in the article/**Supplementary Material**. Further inquiries can be directed to the corresponding authors.

## Author contributions

JW: Data curation, Writing – review & editing, Funding acquisition, Writing – original draft. YY: Visualization, Writing – original draft, Writing – review & editing. XW: Writing – review & editing. RZ: Writing – review & editing, Methodology, Resources, Supervision. QY: Methodology, Resources, Writing – review & editing. FL: Writing – review & editing. GL: Writing – review & editing. HY: Methodology, Writing – review & editing. CG: Methodology, Writing – review & editing. KQ: Writing – review & editing. QW: Resources, Writing – review & editing. SW: Resources, Writing – review & editing. SZ: Funding acquisition, Methodology, Supervision, Writing – review & editing.
